# Glyconanomaterials for Human Virus Detection and Inhibition

**DOI:** 10.3390/nano11071684

**Published:** 2021-06-26

**Authors:** Noelia Losada-Garcia, Carla Garcia-Sanz, Alicia Andreu, Trinidad Velasco-Torrijos, Jose M. Palomo

**Affiliations:** 1 Department of Biocatalysis, Institute of Catalysis (CSIC), Marie Curie 2, 28049 Madrid, Spain; n.losada@csic.es (N.L.-G.); carlagarciasanz@gmail.com (C.G.-S.); aliandreuvillas@gmail.com (A.A.); 2 Department of Chemistry, Maynooth University, Maynooth, W23VP22 County Kildare, Ireland; trinidad.velascotorrijos@mu.ie

**Keywords:** glycan, nanomaterial, glycoconjugates, nanoparticles, virus, coronavirus, SARS-CoV-2, biosensor, antiviral drug

## Abstract

Viruses are among the most infectious pathogens, responsible for the highest death toll around the world. Lack of effective clinical drugs for most viral diseases emphasizes the need for speedy and accurate diagnosis at early stages of infection to prevent rapid spread of the pathogens. Glycans are important molecules which are involved in different biological recognition processes, especially in the spread of infection by mediating virus interaction with endothelial cells. Thus, novel strategies based on nanotechnology have been developed for identifying and inhibiting viruses in a fast, selective, and precise way. The nanosized nature of nanomaterials and their exclusive optical, electronic, magnetic, and mechanical features can improve patient care through using sensors with minimal invasiveness and extreme sensitivity. This review provides an overview of the latest advances of functionalized glyconanomaterials, for rapid and selective biosensing detection of molecules as biomarkers or specific glycoproteins and as novel promising antiviral agents for different kinds of serious viruses, such as the Dengue virus, Ebola virus, influenza virus, human immunodeficiency virus (HIV), influenza virus, Zika virus, or coronavirus SARS-CoV-2 (COVID-19).

## 1. Introduction

Viruses are among the most infectious pathogens, responsible for the highest number of deaths worldwide. Although the pathogenic mechanisms of viruses are diverse, all existing viruses need a host to maintain their existence [1]. The complex glycans attached on the surface of viral envelope proteins (up to half of the molecular weight of these glycoproteins) helps the pathogen elude recognition by the host immune system [2,3] altering the host’s ability to generate an effective adaptive immune response [4] or improving infectivity [5]. Although the innate immune system has evolved a range of strategies for responding to glycosylated pathogens, mutations in such proteins (virus variants) could impact by creating new or removing existing locations of the glycans (glycosites) on the surface antigens [6,7].

Viral infections result in millions of deaths and huge economic losses annually. In recent years, important examples are the viruses Ebola, Zika, SARS, MERS or recently the actual pandemic SARS-CoV-2 coronavirus (which have infected more than 140 million and killed more than 3 million people so far (data from 21 April 2021) [8].

This means that novel systems for rapid and efficient detection of viruses are critical to control the infection spread. From the last decade, nanotechnology has signified an important advance in the development of nanomaterials for detection devices [9,10,11,12]. The fabrication of biosensors based on antibodies, proteins, or biomolecules has been highlighted as a key element for detection of the virus [13].

Glycan molecules have been demonstrated to have a very important role in many biological recognition processes [14]. Indeed, different glycomolecules such as heparin derivatives or sialic acids derivatives have shown antiviral activity [15].

**Figure 1:**
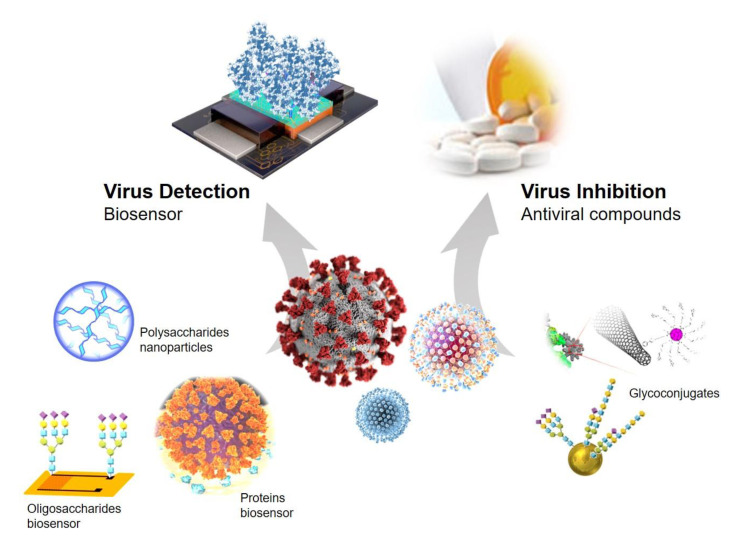
Different glyconanomaterials strategies for application in viral diseases.

Therefore, in this review we summarized the recent advances in the fabrication of novel functionalized glyconanomaterials, evaluating the effect of different glycomolecules as biomarkers in the recognition of human viruses and the important role in the detection of viral glycoproteins in relevant cases such as the Dengue virus, Ebola virus, human immunodeficiency virus (HIV), influenza virus, Zika virus, and coronavirus SARS-CoV-2 as well as their novel application as potential antiviral agents. Different sections will be focused on proteins, oligosaccharide-functionalized nanomaterials, and glyconanoparticles as biosensors for virus detection and glyconanoconjugates for virus inhibition (Figure 1). Another important kind of virus detection method namely antibody-based was described in a recent review article [16].

## 2. Proteins or Oligosaccharides Immobilized in Nanomaterials for Glycoprotein-Virus Detection

### 2.1. Proteins Immobilized in Nanomaterials

One of the most promising strategies for biosensor devices to detect viruses is based on protein-based biosensors [17,18,19,20].

In such cases, some of the main examples using nanomaterials involve the functionalization with lectins, proteins which specifically interact with glycans from glycoproteins. In particular, Concanavalin A (ConA) is a well-known lectin, which binds specifically α-D-mannosyl and α-D-glucosyl residues [21], which are found in the glycoproteins from the viral capsid.

Thus, Oliveira and coworkers [18] developed a biosensor based on cysteine (Cys), zinc oxide nanoparticles (ZnONPs) and Concanavalin A lectin (ConA) to differentiate between arbovirus infections. These have become a major global health problem due to recurrent epidemics [22] and nonspecific clinical manifestations have been developed. The reproducibility, sensitivity and specificity of the sensor for Dengue virus type 2 (DENV2), Zika (ZIKV), Chikungunya (CHIKV), and Yellow fever (YFV) were evaluated.

**Figure 2:**
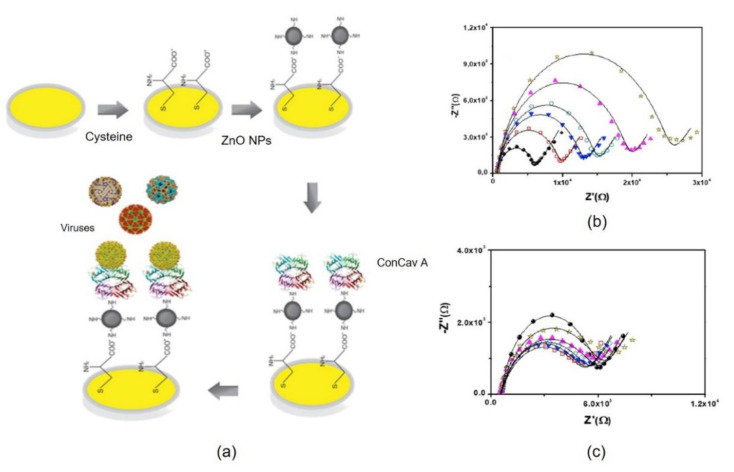
(**a**) Schematic representation of the surface modification. (**b**,**c**) Nyquist diagram of the sensor system (•) and serial dilutions of arboviruses 1:50 (□); 1:40 (∇): 1:30 (⋄); 1:20 (Δ); 1:10 (*) of the virus. (**b**) ZIKV, (**c**) DENV2. Figure adapted from ref. [18].

Differences in viral envelope are an essential feature of immune response differentiation after arbovirus infection. The structural components of the viral envelope can be used to identify and differentiate arboviruses [23]. Therefore, the lectin ConA was used in this work for the identification and differentiation of carbohydrates in the viral cover. Atomic force microscopy measurements confirmed the modification of the electrode surface and revealed a heterogeneous topography during the biorecognition process (Figure 2). To characterize the biosensor, they used cyclic voltammetry (CV) and impedance spectroscopy (EIS), showing a linear response at different concentrations of the arboviruses studied. This study demonstrated that ConA recognizes the structural glycoproteins of DENV2, ZIKV, CHIKV, and YFV (Figure 2).

The degree of affinity of the sensor to the virus decreased from ZIKV > DENV2 > CHIKV > YFV. The sensor shows the highest response to ZIKV due to the interaction of ConA with specific carbohydrate residues present in the structural glycoprotein E of ZIKV (Figure 2b). Also, DENV2 showed a high interaction with ConA due to the presence of glucans on its surface (Figure 2c). This binding between ConA and viral glycoproteins reflects the interaction of the biomolecule with carbohydrate residues present in the glycan monomer Ans154 exposed on the surface of DENV2 and ZIKV Virus [24]. These results support the use of the proposed system for the development of biosensors for arbovirus infections.

Another approach in the design of protein-based biosensors focuses on the use of proteins mimicking the carbohydrate-binding receptor of the virus.

**Figure 3:**
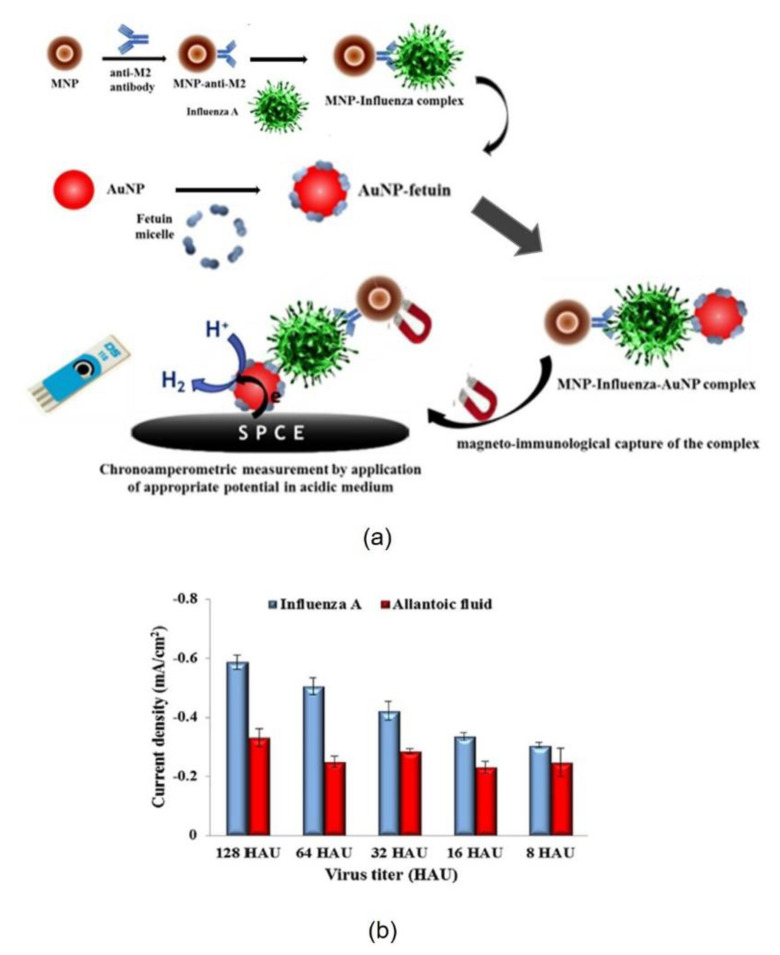
(**a**) Schematic illustration of the strategy used to develop the gold nanoparticle-based chronoamperometric magnetoimmunosensor for influenza virus; (**b**) Diagrams correspond to the response of the magnetoimmuno assay to various influenza virus titers ranging from 8 HAU to 128 HAU (blue) and to various concentrations of non-infected allantoic fluid in 1M HCl solution (red). Reprinted with permission from reference [19]. Copyright 2018 Elsevier.

Fetuin-A (FetA, alpha-2-HS-glycoprotein) is a 64-kDa glycoprotein that is found in relatively high concentrations in human serum and recently has been used for the new selective nanobiosensor developed for influenza virus [19]. The strategy is based on a gold nanoparticle chronoamperometric magnetoimmunosensor for the influenza A H9N2 virus (Figure 3). This method is performed in two steps using two specific influenza receptors. The first, anti-matrix protein 2 (M2) antibody, was bound to magnetic iron nanoparticles (MNP) and used for the isolation of the virus from allantoic fluid. The second biomolecule, Fetuin A (alpha-2-HS-glycoprotein), was bound to an electrochemical detectable gold nanoparticle (AuNP) tag, and it was used to detect the adherence advantage of the virus from the fetuin A hemagglutinin interaction (Figure 3). The complex formed by MNP-Influenza virus-AuNP was isolated and treated with an acid solution, then the collected gold nanoparticles were deposited on a screen-printed carbon electrode (Figure 3a). AuNPs catalyze the reduction of hydrogen ions in an acid medium while applying an appropriate potential, and the current signal generated was proportional to the virus titer. Finally, the chronoamperometric detection of the virus was carried out indirectly by quantifying the AuNPs, allowing the rapid detection of influenza virus A (H9N2) with a titer of less than 16 HAU (Figure 3b). This detection system is able to distinguish between virus influenza A and B by taking advantage of the use of anti-M2 antibodies that specifically recognize influenza A virus.

Therefore, this could be a potential diagnostic sensor for clinical applications, because of the advantages of speed, the use of very small sample volumes, and its suitability for the selective determination of the virus from complex real samples.

**Figure 4:**
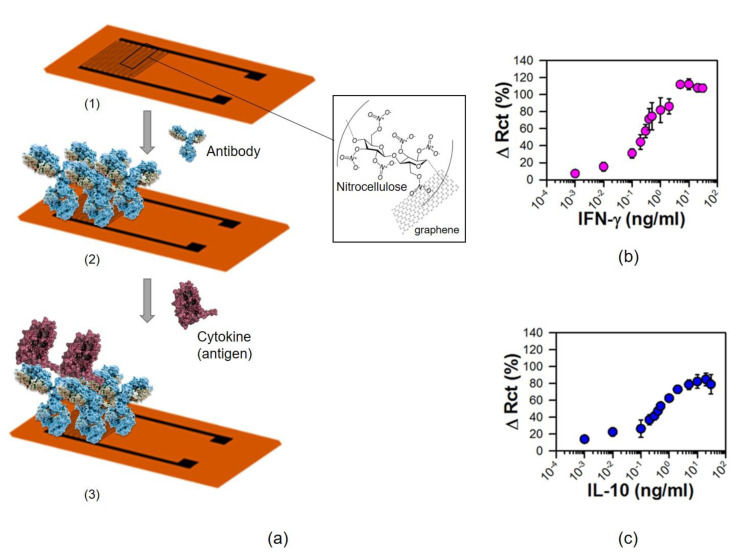
(**a**) Schematic representation of fabrication and biofunctionalization of the IDE. (1) AJP graphene IDE on a polyimide (Kapton) sheet. (2) Antibodies selective to IL-10 or IFN-γ immobilized on functionalized graphene. (3) Incubation with antigen. (**b**,**c**) IFN-γ and IL-10 detection using graphene IDE sensors. Figure adapted from ref. [20].

Another strategy for detection of viral infection involves the development of an imm unosensor capable of monitoring cytokines [20]. Cytokines (e.g., interferons, interleukins, etc.) are small proteins (5–20 kDa) important in cell signaling as immunomodulating agents, which are secreted by different immune cells for fighting off infections (Figure 4). Thus, the strategy developed by Hersam, Claussen, and coworkers [20] is based on a flexible graphene interdigitated electrode (IDE) printed with aerosol jet printed graphene (AJP) for electrochemical bioselection (Figure 4a). This system was developed to detect two different cytokines: interferon gamma (IFN-γ) and interleukin 10 (IL-10).

A graphene-nitrocellulose ink was printed on a flexible polyimide film (Kapton) in an IDE pattern by aerosolizing graphene and depositing the aerosol mist in highly focused lines. The electrode was chemically modified to generate carboxylic groups as functional groups on the surface for covalent immobilization of anti-bovine antibody (IFN-γ or IL-10) which was then used for detecting the corresponding IFN-γ and IL-10 cytokines (Figure 4a). The system showed a very low detection threshold, with a detection limit of 25 pg/mL for IFN-γ and 46 pg/mL for IL-10 (Figure 4b,c). These detection ranges cover the detectable IFN-γ concentration ranges in the clinical disease stage and fit the newly infected stage to clinical disease (in case of IL-10 concentration ranges) for example of Johne’s disease (a chronic enteritis of ruminants caused by M. paratuberculosis). Furthermore, the biosensor was highly selective towards IFN-γ or IL-10 with negligible cross-reactivity with each other and similar cytokines (i.e, IL-6). Therefore, this example is an interesting example in term of application of glyconanomaterials which can be extended to other current diagnosis systems based on antibody-methods [16] for the indirect detection of different viruses such as HIV, coronavirus, or even other diseases [25].

### 2.2. Oligosaccharides Immobilized in Nanomaterials

Most viruses recognize glycans on glycoproteins and glycolipids of the mammalian cells as receptors [26], especially heparin sulfate or sialic acid containing oligosaccharides. Thus, novel nanobiosensors have focused on materials functionalized with these glycans [27,28]. In particular, heparin has been successfully used as analog of heparan sulfate in a biosensor as a biorecognition element in place of the traditional antibody for Dengue virus during infection of Vero cells and hepatocytes [29].

**Figure 5:**
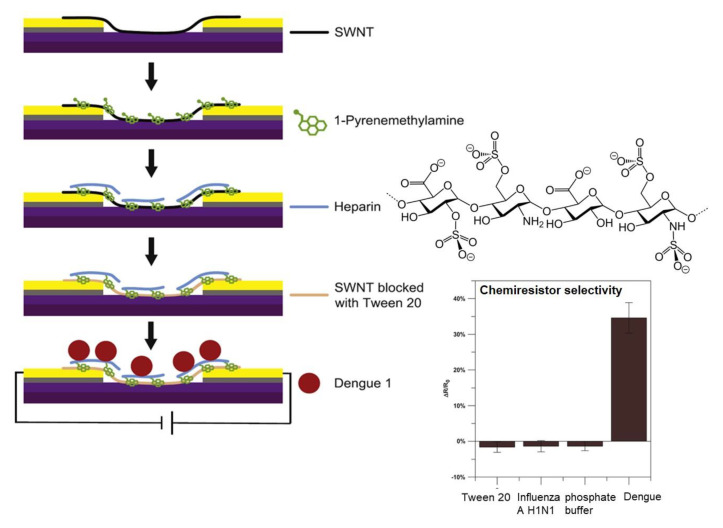
Glyconanomaterial chemiresistor for detection of Dengue virus. Figure adapted from ref. [27].

Thus, Yates and coworkers [27] developed a new electronic biosensor based on a single-walled carbon nanotube (SWNT) lattice chemoresistive transducer that is functionalized with heparin, for the rapid, ultrasensitive, and low-cost detection of the whole Dengue virus (DENV) (Figure 5).

Carbon nanotubes were functionalized with pyrenemethylamine by π–π stacking interaction of the pyrene moiety and the SWNT. Then chemical modification was performed to incorporate heparin to the surface. This oligosaccharide is highly sulfated, having the highest negative charge density of any known biological macromolecule, whereas DENV envelope protein has positively charged β-strands located within a well-conserved binding pocket for electrostatic interaction with receptor sulfate groups. Therefore, interaction between the glycan and virus is detected by the resistance on p-type SWNTs. This method was highly selective since a functionalized sensor responded to the presence of DENV but not to influenza H1N1 (Figure 5). A limit of detection of ~8 DENV/chip was obtained with only 10-min incubation of a 10 μL sample.

However, actually the most important developments in glycan-sensors for virus detection have been focused on the coronavirus SARS-CoV-2, responsible for the pandemic respiratory disease COVID-19 (starting in December 2019) [30], which to date has caused the death of more than 3 million people worldwide (up to 28 April 2021).

**Figure 6:**
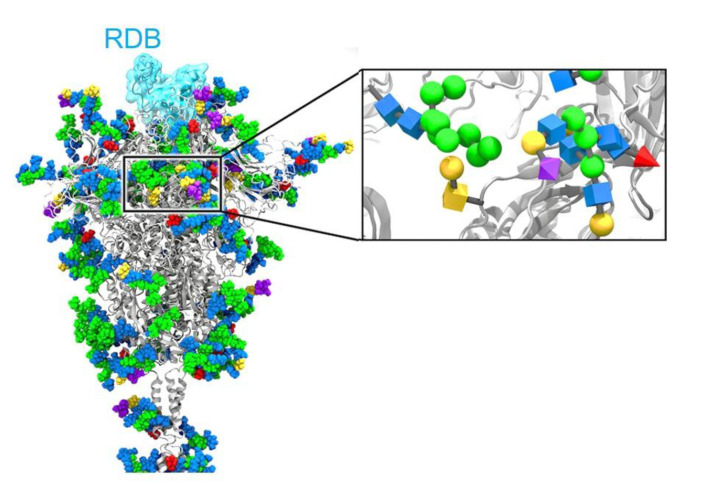
Glycosylated full-length model of the SARS-CoV-2 S protein in the open state where the RBD in the “up” state is highlighted with a transparent cyan surface. N-/O-glycans are shown in Van der Waals representation, where GlcNAc is colored in blue, mannose in green, fucose in red, galactose in yellow, and sialic acid in purple (Magnified view of the S protein stalk glycosylation). Reprinted with permission from reference [32]. Copyright 2020 American Chemical Society.

In this context, sialic acids were determined as a receptor (or recognition motif) for the glycoproteins from the coronavirus, especially the spike protein [31]. In SARS-CoV-2 virus, the spike protein is extensively glycosylated, with particular importance for mannose and sialic acid (Figure 6) [32].

**Figure 7:**
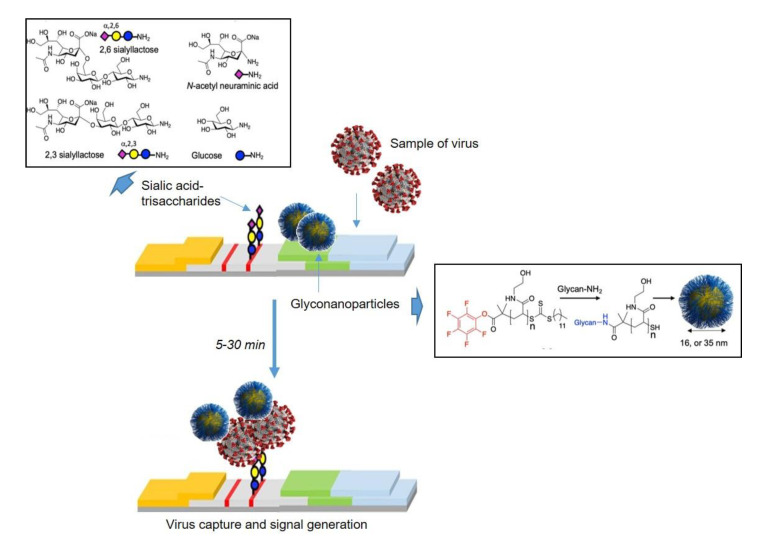
Design concept for glycolateral flow devices. Figure adapted from ref. [28].

Thus, recently Gibson and coworkers [28] described the synthesis of a glyconanomaterial formed by polymer stabilized, multivalent gold nanoparticles bearing sialic acid derivatives for the interaction with the spike glycoprotein from SARS-CoV-2. They found that sialic acid (α,N-acetyl neuraminic acid) binds to the spike glycoprotein and subsequently they could exploit this interaction as the detection unit in a prototype lateral flow rapid diagnostic, which would not require centralized infrastructure. In this strategy, the glycan was immobilized (as a BSA-glycoconjugate) on the test strip and also in the mobile phase onboard gold nanoparticles, providing multivalence (and hence affinity) for dissecting SARS-CoV-2 binding and for the LFD (lateral flow device) (Figure 7).

Herein, the nanoparticle design is based on telechelic polymer tethers which conjugate the glycans (sialic acids), by the displacement of an ω-terminal pentafluorophenyl (PFP) group, and immobilization onto gold particles via an α-terminal thiol. In the next reaction step, poly (*N*-hydroxyethyl acrylamide), PHEA, was chosen as the polymer to give colloidally stable particles (Figure 7) [33].

In contrast, amino-glycans were synthesized by reduction of the anomeric azides and later conjugated to the PHEAs by displacement of the PFP group. Hereinafter, polymers were assembled onto citrate-stabilized gold nanoparticles. Characterization analysis revealed that 35 nm sialyl-lactose particles were the most stable ones and were selected to perform glycan-binding assays. This study replicated a lateral flow situation using protein expressed mammalian cells (HEK) to ensure glycosylation. The assays revealed that in this system sialic acid gave the strongest response and consequently was taken forward. With the successful identification of sialic acid as a target ligand, its application as the capture unit in lateral flow was examined. It is important to highlight that the performance does not only depend on the affinity of the capture ligand but also on the flow of the particles. For this reason, “half” lateral flow assays were established to optimize the particles. The negative test line was (commercial) 2,3-silyllactose-BSA, which the nanoparticle should not bind to (to avoid false positives). The positive control was immobilized SARS-CoV-2 and the sialic acid-AuNPs 35 nm nanoparticles flowed over them. The lateral flow devices could clearly detect the virus-like particles at a concentration of 5 µg/mL protein. Sialic acid particle system had a clear preference for SARS-CoV-2, demonstrating selectivity in this glyconanoparticle system. This data supports the notion that the terminal sialic acid is the key binding motif. Therefore, this system can detect the spike glycoprotein from SARS-CoV-2 virus under 30 min, thus being interesting for the creation of rapid point-of-care diagnostics in a format which requires no infrastructure and limited training.

Other glycans with a relevant role in cell communication, signaling and adhesion, also acting as immuno-modulators during infectious diseases are phosphoglycans such as glycosylphosphatidylinositol (GPI) [34]. This molecule has been successfully used for detection of malaria and other diseases [35], by detection of anti-glycan antibodies. This method also holds great potential for the detection of IgG antibodies related to other multiple medical conditions characterized by overexpression of antibodies, for example SARS-CoV-2 [36].

**Figure 8:**
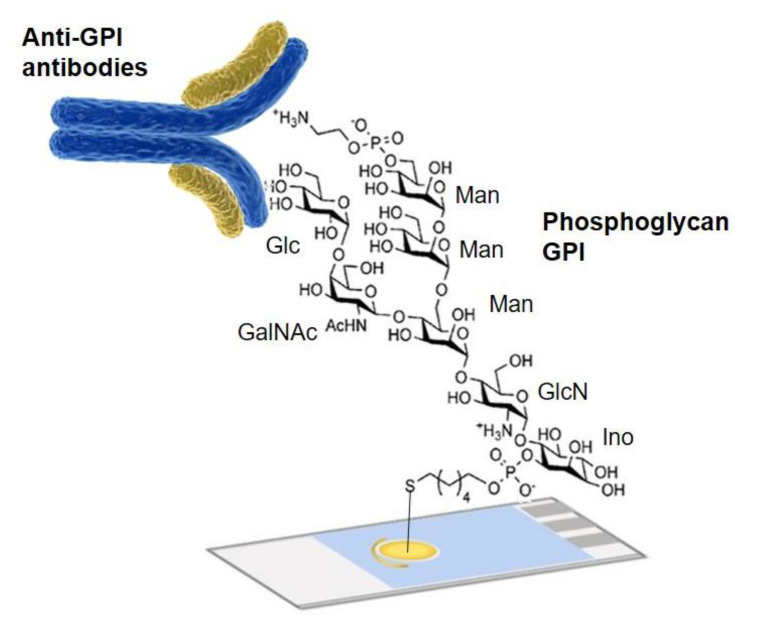
Glycosensor device based on immobilized phosphoglycan. Figure adapted from ref. [37].

Thus, recently a glycobiosensor based on synthetic GPI was developed by Orozco and coworkers [37] (Figure 8). The system consists of a simple label-free electrochemical biosensor developed for monitoring anti-glycan IgG antibodies in serum from toxoplasmosis seropositive patients. The synthetic GPI consisted of a lipid, a phospho-myo-inositol (Ino), a glucosamine (GlcN), three mannoses (Man), and a phosphoethanolamine (PEtN) forming the EtNP-6Man-α-(1 → 2)-Man-α-(1 → 6)-Man-α-(1 → 4)-GlcN-α-(1 → 6)- Ino-phospholipid glycolipid, having an α-Glc-(1 → 4)-β-GalNAc side branch at the O4-position of the first mannose (Figure 8) [34,35,38].

This phosphoglycan (bioreceptor of *T. gondii*) was attached to screen-printed gold electrodes (SPAuEs) through a linear alkane thiol phosphodiester and the interaction with antibodies was detected and quantified by electrochemical impedance spectroscopy (EIS). No significant increase in the Rct (charge transfer resistance) value was observed when the GPI-electrode was incubated with the non-reactive serum whereas an increase was clearly observed with the reactive serum.

Viral infections are associated with around 12% of cancers. Indeed, viruses such as Hepatitis B and C viruses, and human papilloma virus have been shown to contribute to the development of human cancers [39]. Viruses may induce sustained disorders of host cell growth and survival through the genes they express, or may induce DNA damage response in host cells, which in turn increases host genome instability. Moreover, they may induce chronic inflammation and secondary tissue damage favoring the development of oncogenic processes in host cells.

Therefore, another strategy associated with detection of immunoresponses involves the fabrication of oligosaccharide nanoparticles in antitumor therapy [40,41]. Dextran modified glyconanoparticles have been developed in this sense. Nanoparticles such as chitosan cationic nano/microspheres were reported by Pyrc, Szczubiałka and coworkers [42] for selective adsorption of viral particles from aqueous suspensions, which can be useful for the removal of coronaviruses and water purification. *N*-(2-hydroxypropyl)-3-trimethyl chitosan (HTCC) cationic nano/microspheres were prepared by crosslinking of chitosan with genipin, followed by reaction with glycidyltrimethyl-ammonium chloride (GTMAC). After cytopathic and PCR analyses, these glycoparticles adsorbed in a strong and selective way human coronavirus NL63 (HCoV-NL63).

## 3. Glyconanoconjugates as Novel Antiviral Drugs

Glycomolecules conjugated with nanostructures, such as nanoparticles have been also investigated for multiple applications, in particular for novel antiviral therapy.

**Figure 9:**
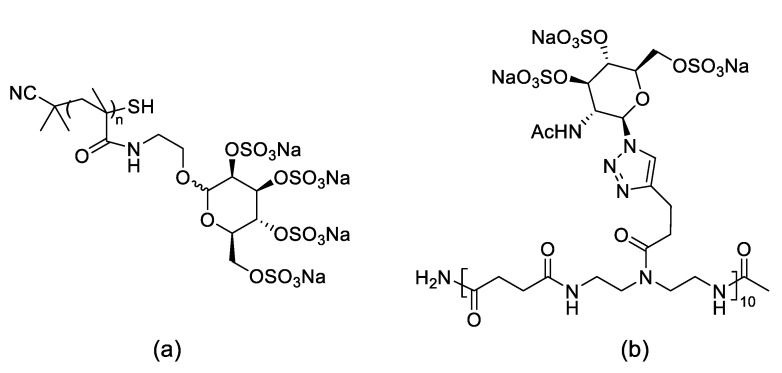
Chemical structure of synthetic sulfated glycomimetic polymers. (**a**) and glycol-oligomers. (**b**) with antiviral activity.

In this sense, sulfated glycopolymers such as heparin and carrageenin have been reported to interact with several types of viruses. However, inherent heterogeneity and toxicity of these naturally occurring glycopolymers may hamper clinical applications [43]. To overcome some of these challenges, Schelhaas and co-workers [44] evaluated the potential of highly sulfated polymeric glycomimetics as inhibitors of viral binding and infection. Glycomimetic were prepared by controlled reverse addition–fragmentation chain-transfer (RAFT) polymerization of methacrylamide-monosaccharide conjugates to give glycopolymers (Figure 9a). Solid-phase polymer synthesis (SPPoS) was employed for the synthesis of glycol-oligomers (up to 10 units, Figure 9b). Both long and short chain poly-glycomimetics were subsequently sulfated. It was found that the glycopolymers were efficient inhibitors of a modified human papillomavirus (HPV16) infection with a potency comparable to carrageenan. Shorter oligomers reduced infection up to 90% at higher concentrations, however the longer glycol-oligomers were less efficient. A study of the mechanism of action of these glycoconjugates using fluorescently labeled HPV16 revealed that while glycopolymers blocked binding and entry of viral particles to host cells, the glycol-oligomers allowed binding but not infection, indicating that infection occurred at a later stage. Remarkably, the sulfated glycopolymers prevented HPV16 infection also in an in vivo mice model. In addition, these glycopolymers blocked infection by other human pathogenic viruses such as HSV-1 (Herpes Simplex Virus), MCPyV (Merkel Cell Polyomavirus), and IAV (Influenza A Virus) with a range of antiviral efficacies. These observations highlight the potential of these glycomimetics as broad spectra antiviral compounds.

The recognition between cell surface glycans and carbohydrate binding proteins in pathogens and host cells is often the first stage of many infectious processes. Blocking these interactions provides a strategy to inhibit pathogen adhesion and subsequent infection. Importantly, this approach can prevent the virus from mutating and developing resistance [45]. DC-SIGN (dendritic cell-specific intercellular adhesion molecule-3-grabbing non-integrin) is a C-type lectin found on the surface of dendritic cells that recognizes mannosylated and fucosylated glycans. DC-SIGN is a key pathogen receptor in the human immune system. Viral infections such as those caused by HIV or Ebola are mediated by DC-SIGN recognition. For this reason, DC-SIGN is an important target for the design of glycoconjugates with antiviral activity [46].

**Figure 10:**
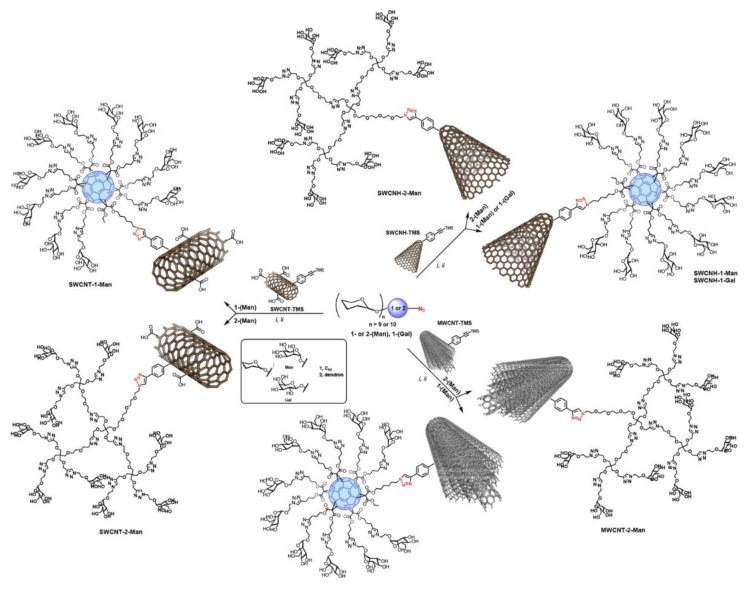
Schematic representation of the chemical modification of single wall carbon nanotubes (SWCNTs), multiwall carbon nanotubes (MWCNTs), and single wall carbonnanohorns (SWCNHs) using the CuAAC “click chemistry” methodology to introduce glycodendrons and glycofullerenes. Reprinted with permission from reference [47]. Copyright 2018 American Chemical Society.

In this sense, recently Martin and coworkers [47] synthesized different novel glyconanoconjugates based on functionalized single wall carbon nanotubes (SWCNTs), multiwall carbon nanotubes (MWCNTs), and single wall carbon-nanohorns (SWCNHs) using the CuAAC “click chemistry” methodology (Figure 10). These carbon-based nanostructures provided scaffolds of different rigidity, morphology and three-dimensional shapes. Chemical modification with glycodendrons and glycofullerenes provided multivalent mannose presentations that mimic the glycans at the viral capside. Binding of these glycoconjugates to DC-SIGN would thus prevent interaction with the virus. The antiviral activity was tested using an artificial Ebola virus assay in which the inhibition of the infection process of DC-SIGN expressing cells was evaluated in the presence of these glyconanostructures at different concentrations.

The results showed that both the nanocarbon platforms (SWCNTs, MWCNTs or SWCNHs) and the multivalent glycan display (glycodendrimers or glycofullerens) strongly influenced the glyconanomaterials’ ability to interact with DC-SIGN and prevent viral infection: glycoconjugates based on MWCNTs functionalized with glycofullerenes were found to be the most potent inhibitors, with IC_50_ values as low as 0.37 μg/mL.

**Figure 11:**
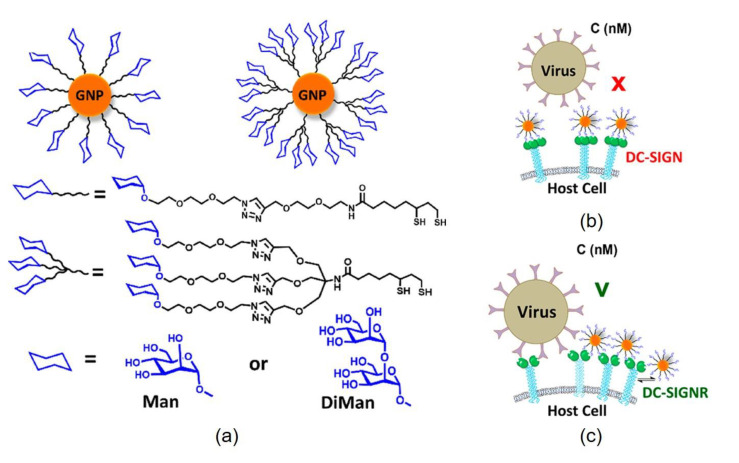
(**a**) Schematic representation of glycan-GNPs coated with lipoic acid containing terminal disaccharide α-mannose-α-1,2-mannose or three terminal α-1-mannose orα-1-mannose-α-1,2-mannose. (**b**) DC-SIGN engages in simultaneous binding with glycan-GNPs, leading to high affinity and potent inhibition of EBOV-GP driven infection. (**c**) DC-SIGNR and glycan-GNPs interactions occur through intercross-linking, leading to lower affinity and low inhibition of EBOV-GP driven infection.

Gold nanoparticles (GNPs) displaying glycans have been extensively studied as multivalent glycomaterials for sensing and biomedical applications [48]. Zhou and co-workers [49] reported GNPs coated with lipoic acid containing terminal α-mannose or the disaccharide α-mannose-α-1,2-mannose (Figure 11) These modified GNPs were of similar size and glycan display to the HIV surface glycoprotein that binds DC-SIGN, leading to HIV infection. Thus, these glycan-GNPs were evaluated as probes to study the binding to DC-SIGN and closely related DC-SIGNR using a novel method based on the fluorescent quenching properties of GNPs. This study revealed that densely coated mannosylated GNPs bind these lectins with high affinity (dissociation constants, Kd, in the nanomolar range), with DC-SIGN binding with higher affinity. Importantly, the authors were able to establish that DC-SIGN engaged in simultaneous binding with glycan-GNPs, while DC-SIGNR and glycan-GNPs interactions occur through intercrosslinking. The correlation between glycan-GNPs affinity for the lectins and the assembly mechanism is relevant to predict how effective these glycoconjugates would be in viral neutralization. In fact, pretreatment of cells expressing DC-SIGN with glycan-GNPs exposed to an artificial model of Ebola virus (EBOV-GP) prevented the inhibition of viral gene transduction in a dose dependent manner: dimannoside glycoconjugate shown in Figure 11a has an IC50 value as low as 95 ± 17 pM, making it the most potent glycoconjugate inhibitor against EBOV-GP driven infection reported to date (Figure 11b). On the other hand, glycan-GNPs, which had lower affinity for DC-SIGNR, were also much less effective in blocking DC-SIGNR mediated transduction, even at high concentrations (Figure 11c).

## 4. Conclusions

This review emphasized the most recent advances in the design and application of different glyconanomaterials for the detection and inhibition of human viruses. This nanotechnological approach, in which it combines the power of nanomaterials, for example nanoparticles—which have been demonstrated in the last decade to be an important tool for biomedical applications—with glycan molecules—which have a key role in biological recognition systems—opens up timely a future way forward in biomedicine. Advantages of this methodology are for example high sensitivity and efficiency, whereas a potential disadvantage in the case of therapy application is the possible cytotoxicity of these nanomaterials.

Novel systems using tailor-made oligosaccharides or particular proteins were emphasized with their highly sensitive, rapid, and economic features for potential industrial diagnosis kits, in particular of high relevance in the case of the actual pandemic (SARS-CoV-2 virus detection).
